# Predicting fluid responsiveness in non-intubated COVID-19 patients

**DOI:** 10.1186/s13613-021-00813-9

**Published:** 2021-01-27

**Authors:** Frederic Michard

**Affiliations:** MiCo, Denens, Switzerland

I read with great interest the article by Caplan et al. [1] about the prediction of fluid responsiveness in spontaneously breathing patients. They nicely showed that when patients perform a standardized respiratory maneuver and the inferior vena cava (IVC) diameter measurements are done precisely 4 cm from the cavo-atrial junction, the magnitude of the IVC respiratory variation becomes highly predictive of fluid responsiveness.

The current COVID-19 pandemic is responsible for a surge of patients hospitalized for hypoxemic pneumonia. Most of them are spontaneously breathing and stay on hospital wards where they receive oxygen. In this context of acute respiratory failure (ARF), excessive fluid administration may increase pulmonary leak, worsen arterial hypoxemia and precipitate tracheal intubation. On the other hand, insufficient fluid administration may promote the development of acute kidney injury and hemodynamic instability. Therefore, in severe COVID-19 cases, predicting fluid responsiveness is recommended by international and WHO guidelines.

At first sight, the method proposed by Caplan et al. [1] is appealing because it is non-invasive and associated with a high predictive value. However, in practice, it may be difficult to implement for several reasons. First, patients with ARF may have difficulties to cooperate and standardize the way they breath. Second, ARF patients are usually sitting in their bed, with significant respiratory abdomen movements, which does not facilitate the assessment of IVC variations from a sub-costal view—not mentioning the fact that obesity is common in COVID-19 patients. Would Caplan et al. [1] recommend their method in this specific clinical context? Third, because echocardiography is increasingly used by non-cardiologists, would Caplan et al. [1] agree with the fact that the level of precision in IVC diameter measurements requested by their method (exactly 4 cm from the cavo-atrial junction) may be a challenge for some operators?

One should also consider that significant respiratory efforts may induce dramatic changes in intrathoracic pressure and venous return. As a result, in spontaneously breathing patients with ARF, respiratory variations in IVC diameter, or in blood pressure (pulsus paradoxus), or in the pulse oximetry waveform, may depend more on the magnitude of respiratory efforts than on the volume status [2]. I noticed that Caplan et al. [1] excluded patients with active expiration from their evaluation. Do they agree that their method may also have limitations in patients making significant inspiratory efforts?

In patients without any clinical and biological signs of shock or acute kidney injury, a fluid restriction strategy is recommended to limit the development of pulmonary edema and prevent ICU admission. In contrast, in patients with hemodynamic instability or biological signs of tissue hypoperfusion, it may be wise to perform a passive leg raising (PLR) maneuver to identify patients who may benefit from receiving fluid (Fig. 1). It is now well established that the lack of increase in blood pressure during a PLR maneuver cannot exclude a significant improvement in blood flow and oxygen delivery. It is therefore recommended to continuously monitor left ventricular stroke volume in order to assess the hemodynamic response to PLR [3].

**Fig. 1:**
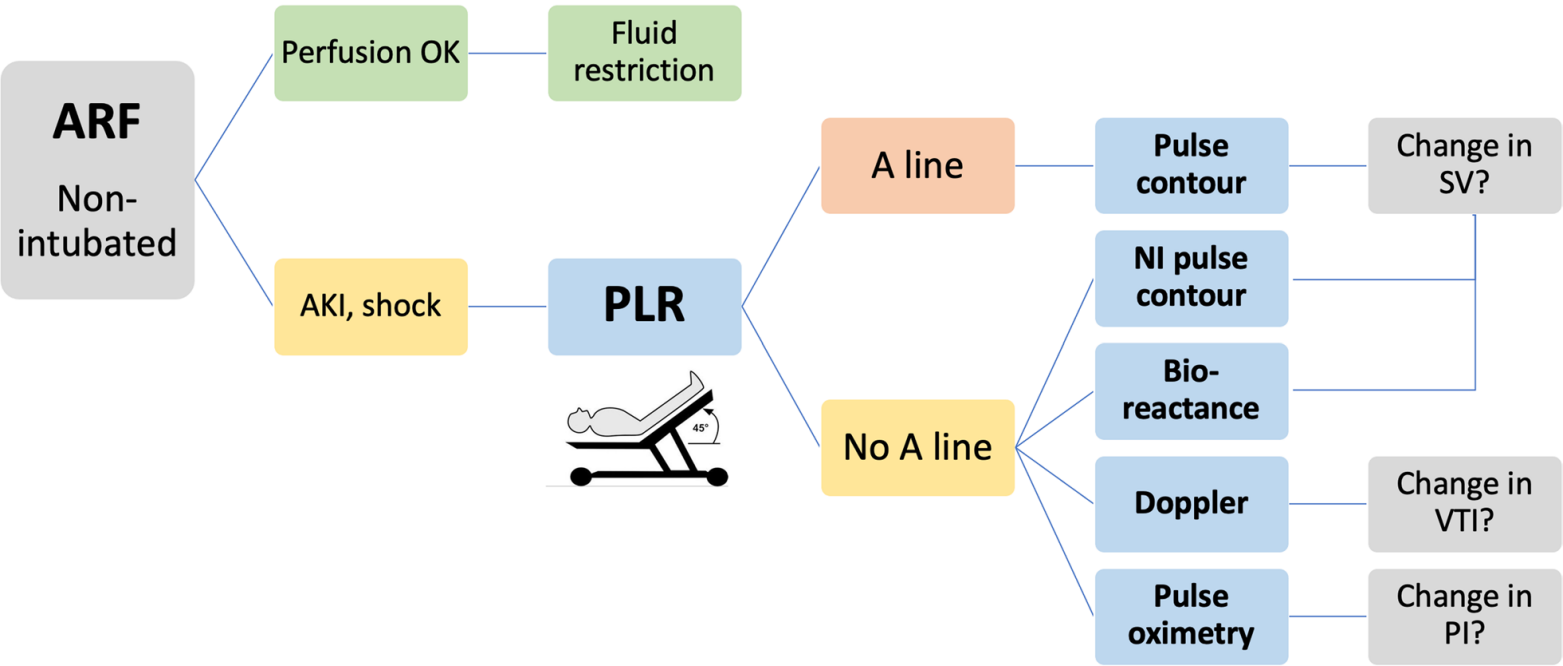
How to predict fluid responsiveness in non-intubated patients with acute respiratory failure (ARF). PLR, passive leg raising maneuver; A line, arterial line; NI, non-invasive; SV, stroke volume; VTI, velocity time integral; PI, perfusion index

In patients who have an arterial catheter in place, a pulse contour technique can be proposed to track changes in stroke volume during the PLR maneuver. In patients who do not have an arterial catheter (most spontaneously breathing patients), there are today basically three options (Fig. 1). The first one is to use a non-invasive cardiac output monitoring system, either a pulse contour technique or a bioreactance method. Both have been shown to be useful to track changes in stroke volume during a PLR maneuver. The second option is to track changes in blood velocity time integral (VTI) using echo-Doppler. It can be done either with a Point Of Care UltraSound (POCUS) device from a thoracic view or with a Doppler probe in the supra-sternal area. In the future, one may also use a wireless Doppler patch positioned on the carotid artery [4].

When none of the above sophisticated systems is available (which is today the case in most hospital wards), one may consider using a pulse oximeter (Fig. 1). Indeed, in addition to SpO_2_ and pulse rate numbers, pulse oximeters display the peripheral perfusion index (PI). The two main determinants of PI are vascular tone and stroke volume. A brief mechanical maneuver like PLR is unlikely to induce significant changes in vascular tone. As a result, it has recently been shown that tracking changes in PI may replace the direct assessment of stroke volume during a PLR maneuver [5].

In summary, although the standardized method described by Caplan et al. [1] may have value to predict fluid responsiveness in calm, cooperative and non-obese patients, it may be difficult to use in ARF patients in real life conditions. With the current surge of hypoxemic COVID-19 patients, clinicians need more than ever simple solutions to rationalize fluid therapy and improve quality of care.

## Data Availability

Not applicable.
